# Nodule-Specific NRF2-Targeted Upregulation in Patients With *KEAP1* Mutations and Familial Nontoxic Multinodular Goiter

**DOI:** 10.1210/clinem/dgae699

**Published:** 2024-10-07

**Authors:** Eijun Nishihara, Shuji Fukata, Mitsuyoshi Hirokawa, Miyoko Higuchi, Mitsuru Ito, Mitsushige Nishikawa, Akira Miyauchi, Michiko Matsuse, Norisato Mitsutake, Yuka Ito, Akira Hishinuma, Takahiko Kogai, Takashi Akamizu

**Affiliations:** Center for Excellence in Thyroid Care, Kuma Hospital, Kobe 650-0011, Japan; Center for Excellence in Thyroid Care, Kuma Hospital, Kobe 650-0011, Japan; Center for Excellence in Thyroid Care, Kuma Hospital, Kobe 650-0011, Japan; Center for Excellence in Thyroid Care, Kuma Hospital, Kobe 650-0011, Japan; Center for Excellence in Thyroid Care, Kuma Hospital, Kobe 650-0011, Japan; Center for Excellence in Thyroid Care, Kuma Hospital, Kobe 650-0011, Japan; Center for Excellence in Thyroid Care, Kuma Hospital, Kobe 650-0011, Japan; Department of Radiation Medical Sciences, Atomic Bomb Disease Institute, Nagasaki University, Nagasaki 852-8523, Japan; Department of Radiation Medical Sciences, Atomic Bomb Disease Institute, Nagasaki University, Nagasaki 852-8523, Japan; Department of Genetic Diagnosis and Laboratory Medicine, Dokkyo Medical University, Tochigi 321-0293, Japan; Department of Infection Control and Clinical Laboratory Medicine, Dokkyo Medical University, Tochigi 321-0293, Japan; Department of Infection Control and Clinical Laboratory Medicine, Dokkyo Medical University, Tochigi 321-0293, Japan; Department of Genetic Diagnosis and Laboratory Medicine, Dokkyo Medical University, Tochigi 321-0293, Japan; Department of Infection Control and Clinical Laboratory Medicine, Dokkyo Medical University, Tochigi 321-0293, Japan; Center for Excellence in Thyroid Care, Kuma Hospital, Kobe 650-0011, Japan

**Keywords:** *KEAP1* germline mutation, multinodular goiter, familial goiter, NRF2-targeted gene

## Abstract

**Context:**

Kelch-like ECH-associated protein 1 (KEAP1) is associated with nuclear factor erythroid-2–related factor 2 (NRF2) and promotes NRF2 degradation in normal conditions. Genetic abnormality in *KEAP1* is a rare disease and presents with familial multinodular goiter.

**Objective:**

This study assessed the clinical and molecular findings concerning nodular formation in the thyroid gland of patients harboring *KEAP1* germline mutations.

**Methods:**

Next-generation sequencing analysis targeting goiter-associated genes was performed on 39 patients with familial multinodular goiter. The expression of NRF2-targeted genes from surgical thyroid specimens of patients with *KEAP1* mutations were analyzed using a whole-transcript expression array and immunohistochemistry.

**Results:**

We found 5 probands with pathogenic heterozygous mutations in *KEAP1* (p.Q86*, p.L136P, p.V411fs, p.R415C, and p.R483H) that had no meaningful concomitance with mutations of other goiter-associated genes at germline and somatic levels. Their common histopathological features showed multinodular goiters in the entire thyroid gland with few degenerative lesions or complications of malignancy and slow proliferation indicating less than 1% at the Ki-67 labeling index. Among 42 NRF2-targeted genes, antioxidant genes were most frequently upregulated (11/12) in the nodule, followed by detoxification genes (6/11). Immunohistochemical analysis showed relatively high expression of glutathione peroxidase 2 and NAD(P)H quinone oxidoreductase 1 (representative NRF2-targeted genes) in the nodules of various patients harboring *KEAP1* mutations.

**Conclusion:**

*KEAP1* germline heterozygous mutations exert excessive NRF2 activity in the thyroid gland and may confer cytoprotective effects even under abundant reactive oxygen species associated with thyroid hormone production, resulting in thyroid hyperplasia with scarce degradation.

Multinodular goiter presents with multifocal hyperplastic/neoplastic lesions, and these nodules are frequently clonal. In the 2022 World Health Organization classification of thyroid neoplasms, multinodular goiter has been introduced in classifying benign tumors; the term *thyroid follicular nodular disease* is based on clonal proliferation (1). Most familial multinodular goiter cases present with an autosomal dominant pattern of inheritance. Indeed, several genetic abnormalities, including *DICER1*, *KEAP1*, and *DGCR8*, have been identified in familial multinodular goiter (2-4).

Kelch-like ECH-associated protein 1 (KEAP1) was originally found to be associated with nuclear factor erythroid-2–related factor 2 (NRF2) (5) and functions as a substrate adaptor protein for a Cul3-dependent E3 ubiquitin ligase complex, with subsequent degradation of NRF2 by the proteasome (6). NRF2 activates the transcription of various cytoprotective genes that enhance cell proliferation (7). Somatic mutations in *KEAP1* in cancer tissues and cancer-derived cell lines are advantageous for cell growth (7). However, germline mutations in *KEAP1* have not been reported in cases of familial cancer; it has been detected in only 2 families with multinodular goiter (3, 8). The thyroid tissue–specific phenotype also applies to *Keap1* knockdown mice that develop diffuse goiter without thyroid nodules or hyperplasia (9). Why *KEAP1* germline mutations are susceptible to a thyroid-predominant phenotype remains unknown.

Oxidative stress or electrophiles inactivate KEAP1 by modifying cysteine residues, which results in decreased ubiquitin activity and nuclear accumulation of NRF2 from the cytoplasm. In thyroid tissues with a *KEAP1* germline heterozygous mutation (R483H), NRF2-positive staining of the nuclei is detected in more than 30% of cells in thyroid nodules and less than 10% of cells in nonnodular parenchyma (8). External stimuli and genetic changes lead to alterations in the cellular localization of NRF2.

In this study, we investigated the clinical and molecular findings associated with 5 different *KEAP1* germline heterozygous mutations, including 4 newly identified mutations.

## Materials and Methods

### Screening of Goiter-associated Gene Variants Using Next-Generation Sequencing

**Figure 1.**
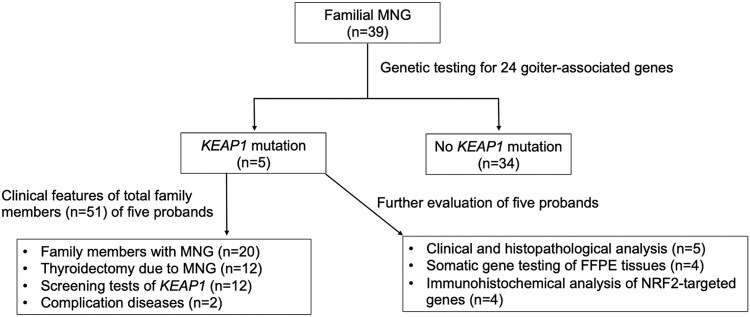
Flowchart of the study population. Abbreviations: FFPE, formalin-fixed paraffin-embedded; MNG, multinodular goiter.

Genetic testing was retrospectively performed on 39 patients with familial multinodular goiter (multinodular goiter detected in a patient and relatives within the second degree of consanguinity) who visited Kuma Hospital between 2014 and 2021 and agreed to genetic testing (Fig. 1). Of the 39 patients, 11 underwent thyroidectomy during their clinical courses. Analysis was performed by next-generation sequencing (NGS) targeting most of the coding exons of 24 goiter-associated genes (Goiter Ampliseq panels A and B, Table 1) using custom primers designed using Ion Ampliseq Designer (Life Technologies), according to the manufacturer's instructions. Briefly, genomic DNA was isolated from whole blood using a QIAamp DNA Blood Mini Kit (Qiagen), and a multiplex polymerase chain reaction of A, 429 and B, 263 amplicons was performed, followed by the addition of IonCode Adaptors using an Ion Chef system (Life Technologies).

**Table 1.** Genes included in goiter Ampliseq

Gene	Chromosome
Panel A	
*NRAS*	Chr1
*TPO*	Chr2
*THRB*	Chr3
*IYD*	Chr6
*SLC26A4*	Chr7
*TG*	Chr8
*RET*	Chr10
*HRAS*	Chr11
*KRAS*	Chr12
*TSHR*	Chr14
*DICER1*	Chr14
*DUOX2*	Chr15
*DUOXA2*	Chr15
*SLC5A5*	Chr19
*KEAP1*	Chr19
Panel B	
*RGS12*	Chr4
*GRPEL1*	Chr4
*WFS1*	Chr4
*APC*	Chr5
*SLC26A7*	Chr8
*WRN*	Chr8
*PTEN*	Chr10
*PRKAR1A*	Chr17
*GNAS*	Chr20
Panel C	
*NRAS*	Chr1
*TPO*	Chr2
*ZNF148*	Chr3
*THRB*	Chr3
*WFS1*	Chr4
GRPEL1	Chr4
*RGS12*	Chr4
*IYD*	Chr6
*KMT2C*	Chr7
*SLC26A4*	Chr7
*BRAF*	Chr7
*TG*	Chr8
*SLC26A7*	Chr8
*ARFGEF1*	Chr8
*WRN*	Chr8
*RET*	Chr10
*PTEN*	Chr10
*HRAS*	Chr11
*KRAS*	Chr12
*TUBGCP3*	Chr13
*TSHR*	Chr14
*DICER1*	Chr14
*DUOX2*	Chr15
*DUOX1*	Chr15
*DUOXA2*	Chr15
*SPOP*	Chr17
*EZH1*	Chr17
*PRKAR1A*	Chr17
*KEAP1*	Chr19
*SLC5A5*	Chr19
*GNAS*	Chr20
*EIF1AX*	ChrX
*KDM5C*	ChrX

To verify somatic mutations in thyroid tumors, DNA was isolated from 4 samples of available formalin-fixed paraffin-embedded (FFPE) thyroid tissues using a QIAamp DNA Mini Kit (Qiagen), and the same procedures as germline mutations for 1414 amplicons were performed by NGS analysis using Goiter Ampliseq panel C.

After enrichment by clonal emulsion polymerase chain reaction on Ion Sphere particles, the barcoded libraries were loaded on an Ion 318 chip, Ion PI chip, or Ion 540 chip, and massively parallel sequencing was conducted on an Ion Torrent PGM sequencer, Ion Proton sequencer, or Ion GeneStudio S5 system with an Ion PGM Hi-Q View Chef kit, Ion PI Hi-Q Chef kit, or Ion 540 Kit-Chef, respectively. We analyzed the raw signal data of NGS using Torrent Suite v.5.2.2 or v.5.4, including adaptor trimming, read alignment to the human genome 19 reference, coverage analysis, and variant calling. We performed variant filtration and annotation using Ion Reporter v.5.0 (Life Technologies) to determine whether the detected sequence variants were known pathogenic mutations or novel variants.

The pathogenicity of 3 missense variants was estimated using computational algorithms, PolyPhen2 (http://genetics.bwh.harvard.edu/pph2/index.shtml), PROVEAN (http://provean.jcvi.org/seq_submit.php), and PANTHER (http://www.pantherdb.org/tools/csnpScoreForm.jsp), and evaluated as “disease-causing or deleterious.” The frequency of each variant in the general population was evaluated using gnomAD (https://gnomad.broadinstitute.org/)

### Affymetrix Whole-Transcript Expression Array

Thyroid specimens resected from the nodule and nonnodular parenchyma (∼100 mg each) available from a patient with a *KEAP1* mutation (L136P, IV-1) were quickly immersed in RNAlater Stabilization Solution (Thermo Fisher Scientific). Total RNA was extracted using the RNeasy Plus Universal kit (Qiagen) according to the manufacturer's instructions. RNA purity and integrity were evaluated using an ND-2000 Spectrophotometer (NanoDrop, Thermo Fisher Scientific) and an Agilent 2100 Bioanalyzer (Agilent Technologies). The Affymetrix Whole-transcript Expression array process was executed according to the manufacturer's protocol (GeneChip Whole Transcript PLUS reagent Kit). Complementary DNA was synthesized using a GeneChip WT amplification kit, according to the manufacturer's instructions. The sense complementary DNA was then fragmented and biotin-labeled with terminal deoxynucleotidyl transferase using a GeneChip WT Terminal labeling kit. Approximately 5.5 μg of labeled DNA was hybridized to the Affymetrix GeneChip Human Clariom S Array at 45 °C for 16 hours. Hybridized arrays were washed and stained on GeneChip Fluidics Station 450 and scanned on a GCS3000 Scanner (Thermo Fisher Scientific). Signal values were calculated using Affymetrix GeneChip Command Console software. Differentially expressed genes were selected at a *P* value of less than .05 and a threshold of fold change (FC) value ≥2.0 or ≤−2.0.

### Histopathological Evaluation and Immunohistochemistry

After surgical resection, thyroid tissues were fixed in 10% neutral buffered formalin, and the specimens were embedded in paraffin. Serial sections (3-µm thick) were cut from each paraffin block. The sections were stained with hematoxylin-eosin (HE) for light-microscopic examination. Immunostaining for human Ki-67 (MIB1, 1:200; Dako), human thyroglobulin (TG; 1D4, 1:400; Leica Microsystems), human NAD(P)H quinone oxidoreductase 1 (NQO1; A180, 1:400; Santa Cruz), and human glutathione peroxidase 2 (GPX2; EPR8175(2), 1:800; Abcam) was performed using a Leica Bondmax system (Leica Microsystems) and a Bond refine kit (Leica Microsystems), according to the manufacturer's instructions. The Ki-67 labeling index was calculated as the percentage of positively stained nuclei at 400× magnification. Control specimens were obtained from 4 unrelated patients with large multinodular goiters (>100 g), who were negative for antithyroid peroxidase (TPO) and anti-TG antibodies (Abs).

This study was approved by the ethics committee of Kuma Hospital, and informed consent was obtained from the patients and their family members for using their samples in this study.

## Results

### Identification and Clinical Characteristics of 5 Families With a *KEAP1* Germline Mutation

**Figure 2.**
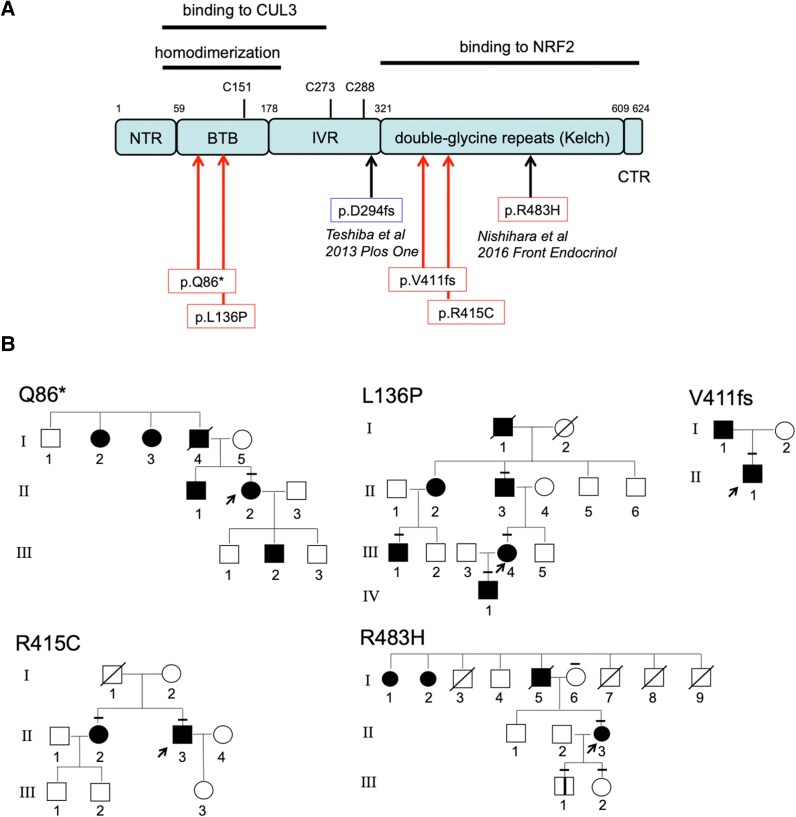
Identifying families with Kelch-like ECH-associated protein 1 (*KEAP1*) mutations. (A) Localization of germline *KEAP1* mutations. The structure is represented by functional regions and corresponding amino acid numbers. Five mutations were identified in our hospital and 1 mutation was reported in another institute. (B) Pedigree of the investigated family. Individuals affected by goiter are indicated by filled symbols. The arrow shows the proband. The bars above each symbol indicate individuals who underwent sequencing analysis for *KEAP1*.

Genetic analysis of 39 patients with familial multinodular goiter (see Fig. 1) identified 4 newly heterozygous variants in germline *KEAP1* (c.256C > T, p.Q86*; c.407T > C, p.L136P; c.1231_1232insG, p.V411fs; and c.1243C > T, p.R415C), in addition to the previously reported variant (c.1448G > A, p.R483H) (8). They were located at the cullin 3- and NRF2-binding regions; 2 were stop codons or frameshifts, and the other 3 were missense mutations (Fig. 2A and 2B). The 3 missense variants were evaluated as “probably damaging or deleterious” (Table 2). Although genetic analysis of family members was limited to almost half (9/20) of the patients with multinodular goiter in 5 families (see Fig. 2B), we judged these variants to be very strong to moderate pathogenic variants, based on the American College of Medical Genetics and Genomics guidelines (10).

**Table 2.** Genetic and clinical characteristics of 5 probands with *KEAP1* variants in familial multinodular goiter

*KEAP1* variants (heterozygous)	p.Q86*	p.L136P	p.V411fs	p.R415C	p.R483H
Frequency (gnomAD)	<0.001%	<0.001%	<0.001%	<0.001%	<0.001%
PolyPhen2 (cutoff: 0.5)	Not subject	Probably damaging (1)	Not subject	Probably damaging (0.997)	Probably damaging (0.994)
PROVEAN (cutoff: –2.5)	Not subject	Deleterious (−6.609)	Not subject	Deleterious (−7.751)	Deleterious (−4.42)
PANTHER (cutoff: –3)	Not subject	Deleterious (−4.17968)	Not subject	Deleterious (−5.05895)	Deleterious (−4.09048)
Sex	Female	Female	Male	Male	Female
Onset of goiter, age in years	13	22	17	37	35
Complication disease	None	None	None	None	Graves disease
Thyroid function	Euthyroid	Euthyroid	Euthyroid	Euthyroid	Euthyroid∼ hyperthyroid
Tg level, ng/mL	1125	4769	736	91	742
TgAb/TPOAb	Negative	Negative	Negative	Negative	Negative (TRAb positive)
Maximum tumor size, mm	39	46	57	47	41
Tumor localization	Bilateral	Bilateral	Bilateral	Bilateral	Bilateral
Thyroidectomy	Done (another hospital)	Done	Done	Done	Done
Thyroid weight, g	No data	No data	690	No data	256

Abbreviations: *KEAP1*, Kelch-like ECH-associated protein 1; Tg, thyroglobulin; TgAb, thyroglobulin antibodies; TPOAb, thyroid peroxidase antibodies.

**Figure 3.**
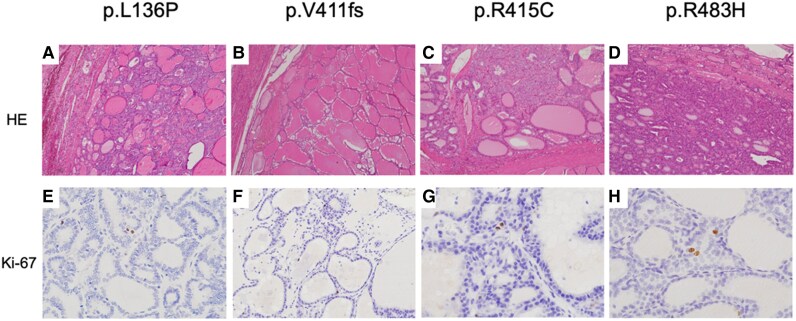
(A-D) Histopathological features and the (E) and (F) Ki-67 labeling index in the thyroid nodules of 4 probands with Kelch-like ECH-associated protein 1 (*KEAP1*) mutations.

Clinical findings on the probands of 5 families with abnormalities of *KEAP1* showed that all variants were heterozygous, and the age of goiter onset ranged between 13 and 37 years. TG levels were elevated, and TGAb and TPOAb levels were negative. All multinodular lesions filled both lobes of the thyroid gland, and the entire thyroid gland was markedly enlarged, which was pathologically confirmed by mixing adenomatous goiter and small follicular adenoma with few degenerative lesions (see Table 2;Fig. 3). The Ki-67 labeling index of all nodules was <1% (see Fig. 3). Next, we evaluated the clinical features of the total number of family members (n = 51) of 5 probands (see Fig. 1). While these 5 families consisted of 20 members with multinodular goiter, 12 patients underwent thyroidectomy based on the surgical indication criteria at our institution (11), 3 of whom underwent multiple surgeries (Figs. 1 and 2B). Thyroid nodules of family members other than the 5 probands had similar pathological findings to those of the probands. A total of 12 family members underwent genetic testing of the *KEAP1* gene, including 9 patients with multinodular goiter and 3 family members without multinodular goiter (see Fig. 2B). All patients with multinodular goiter harbored *KEAP1* mutations. Of the 3 members without multinodular goiter, 2 (R483H I-6 and III-2; see Fig. 2B) harbored no mutation in *KEAP1*, and 1 (R483H III-1; see Fig. 2B) had the same mutation as the proband but had no goiter or nodular lesions at age 22 at follow-up. While no thyroid cancer or neoplastic lesions in other organs were observed in the probands (see Table 2), 2 family members (Q86* I-4 and L136P III-1; see Figs. 1 and 2B) had a history of anaplastic thyroid and esophageal cancers, respectively. The patient case with anaplastic thyroid cancer was not tested for the *KEAP1* mutation, while the patient case with esophageal cancer was confirmed to harbor the *KEAP1* mutation.

Somatic gene testing of 4 FFPE tissues showed that these 4 samples harbored *KEAP1* mutations identical to germline mutation. Moreover, the allele frequency of somatic mutation was greater than 80% in L136P and R483H (Table 3).

**Table 3.** Comparison of variant allele frequency between germline and somatic mutations

Germline					Somatic				
Gene	Amino acid change	Coding	VAF, %	Coverage reference, variant	Gene	Amino acid change	Coding	VAF, %	Coverage reference, variant
*KEAP1*	p.L136P	c.407T > C	48.9	T = 2041, C = 1952	*KEAP1*	p.L136P	c.407T > C	86.8	T = 263, C = 1723
					*RET*	p.Q796*	c.2386C > T	24.6	C = 211, T = 69
*KEAP1*	p.V411fs	c.1231_1232insG	43.8	T = 293, TG = 228	*KEAP1*	p.V411fs	c.1231_1232insG	45.5	T = 1078, TG = 901
*KEAP1*	p.R415C	c.1243C > T	54.0	C = 182, T = 214	*KEAP1*	p.R415C	c.1243C > T	53.2	C = 936, T = 1064
					*DICER1*	p.R1003*	c.3007C > T	7.11	C = 235, T = 18
					*PTEN*	p.G338R	c.493G > A	9.46	G = 1851, A = 145
*KEAP1*	p.R483H	c.1448G > A	48.0	G = 302, A = 279	*KEAP1*	p.R483H	c.1448G > A	82.8	G = 689, A = 3309

Abbreviations: *KEAP1*, Kelch-like ECH-associated protein 1; VAF, variant allele frequency.

### NFR2-targeted Gene Expression in the Thyroid Nodule and Nonnodular Parenchyma in a Patient With Mutated *KEAP1*

A comprehensive gene expression profile in the thyroid nodule and nonnodular thyroid parenchyma of a patient harboring a *KEAP1* mutation (p. L136P) was performed using an RNA microarray. We focused on NRF2-targeted genes that had the binding site antioxidant response element (ARE) at their promoter and enhancer regions and also had experimentally identified functional ARE in humans (12-14). We found 42 NRF2-targeted genes among the approximately 20 000 analyzed genes. The FC values of these NRF2-targeted genes showed that messenger RNA expression of 24 genes was significantly upregulated (2.03 to 496.64) in the thyroid nodule; that of 1 gene was significantly downregulated (−6.75), and the expression of the remaining 17 genes was not significantly different (−1.93 to 1.83; Table 4). Antioxidant genes were most frequently upregulated (11/12) followed by detoxification genes (6/11) in the 4 functional categories.

**Table 4.** RNA microarray analysis of experimentally identified functional NRF2-targeted genes in a case of *KEAP1* mutation (p. L136P)

Gene	FC value*^a^*	Expression		Function
		Nodule	Nonnodule	
*GCLC*	**36**.**41**	15.68	10.49	Antioxidant
*GCLM*	**3**.**80**	10.79	8.86	Antioxidant
*GLRX*	**3**.**17**	10.84	9.17	Antioxidant
*GPX2*	**496**.**64**	14.78	5.82	Antioxidant
*GSR*	**10**.**38**	14.56	11.18	Antioxidant
*PRDX1*	**2**.**40**	16.74	15.48	Antioxidant
*PRDX6*	1.08	14.83	14.72	Antioxidant
*SLC7A11*	**82**.**33**	14.23	7.87	Antioxidant
*SOD1*	**2**.**13**	15.04	13.95	Antioxidant
*SRXN1*	**4**.**29**	10.44	8.34	Antioxidant
*TXN*	**7**.**55**	16.29	13.37	Antioxidant
*TXNRD1*	**18**.**54**	16.43	12.22	Antioxidant
*ABCB11*	−1.88	3.20	4.11	Detoxification
*ABCB6*	**9**.**36**	9.23	6.01	Detoxification
*AKR1C1*	**45**.**84**	17.06	11.55	Detoxification
*AKR1C2*	**95**.**69**	18.00	11.42	Detoxification
*APOA1*	1.15	5.76	5.56	Detoxification
*GSTP1*	**2**.**23**	16.05	14.90	Detoxification
*MGST2*	−1.13	10.70	10.88	Detoxification
*NQO1*	**55**.**47**	16.93	11.14	Detoxification
*NQO2*	−1.20	8.94	9.21	Detoxification
*UGT1A1*	**14**.**70**	7.12	3.24	Detoxification
*UGT2B7*	1.16	3.71	3.50	Detoxification
*BLVRB*	**2**.**62**	13.29	11.90	Heme and iron metabolism
*ETS1*	−1.93	10.04	10.98	Heme and iron metabolism
*FECH*	**2**.**60**	9.81	8.43	Heme and iron metabolism
*FTH1*	1.93	17.73	16.78	Heme and iron metabolism
*FTL*	**2**.**03**	16.39	15.37	Heme and iron metabolism
*HMOX1*	−1.18	6.27	6.50	Heme and iron metabolism
*MT1B*	−1.93	15.15	16.10	Heme and iron metabolism
*ATF3*	−1.37	12.69	13.14	Others
*BACH1*	1.83	12.78	11.91	Others
*GNAI2*	1.46	12.07	11.52	Others
*KRT16*	−1.34	8.24	8.66	Others
*MAFG*	**2**.**32**	7.02	5.81	Others
*MAPT*	−1.11	6.40	6.55	Others
*ME1*	**11**.**75**	13.07	9.51	Others
*PSMA3*	**2**.**88**	12.86	11.34	Others
*PTGS2*	**−6**.**75**	5.15	7.91	Others
*S100A6*	1.64	12.86	12.14	Others
*TBXAS1*	**5**.**31**	10.24	7.84	Others
*TG*	1.04	18.28	18.22	Others

Abbreviations: FC, fold change; *KEAP1*, Kelch-like ECH-associated protein 1; NRF2, nuclear factor erythroid-2–related factor 2.

^*a*^The significant FC values are expressed in bold.

### Immunohistochemical Analysis of Thyroglobulin, NAD(P)H Quinone Oxidoreductase 1, and Glutathione Peroxidase 2 in the Thyroid Gland of Patients With *KEAP1* Mutations

**Figure 4.**
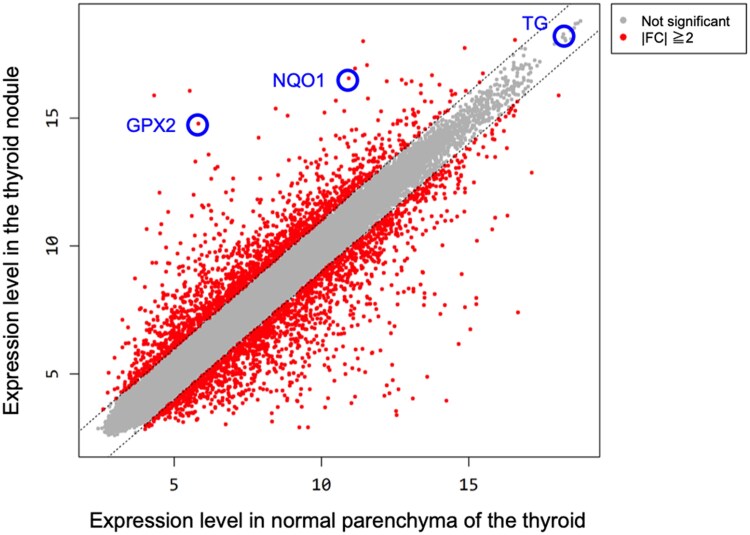
Scatter plots of RNA expression levels. Each gene expression is distributed at the intersection of the thyroid nodular lesion (vertical axis) and normal parenchyma (horizontal axis) of the patient with a Kelch-like ECH-associated protein 1 (*KEAP1*) mutation (L136P). The expression of GPX2, NQO1, and TG is indicated in the blue circle.

**Figure 5.**
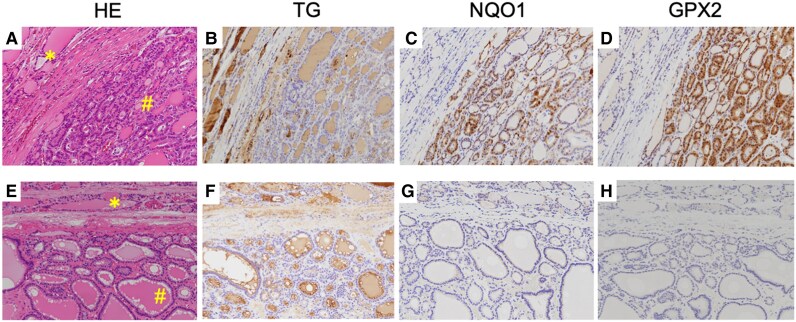
Immunohistochemical detection of NRF2 target genes in the thyroid. (A-D) The upper panels represent a patient with a Kelch-like ECH-associated protein 1 (*KEAP1*) mutation (L136P), and (E-H) the lower panels represent a control patient harboring no *KEAP1* mutation. Histopathological feature of nonnodular parenchyma (*) and thyroid nodules (#) in A and E, hematoxylin-eosin staining; B and F, TG; C and G, NQO1; and D and H, GPX2 expression. The data are representative of 2 experiments with similar results.

**Figure 6.**
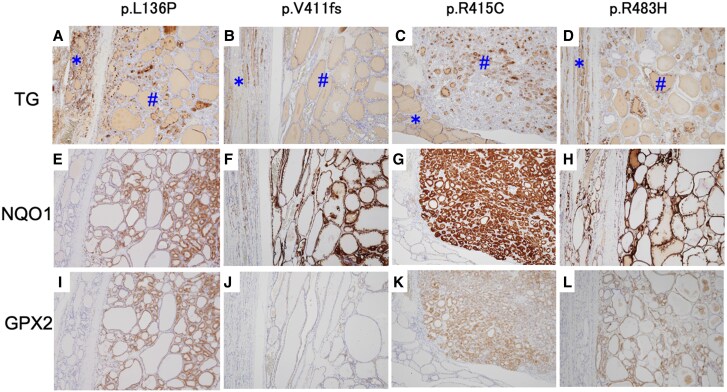
Kelch-like ECH-associated protein 1 (*KEAP1*) mutations and nuclear factor erythroid-2 related–factor 2 (NRF2)-targeted gene expression. (A-D) TG; (E-H) NQO1; and I to L, GPX2 expression in the thyroid of 4 probands with different *KEAP1* mutations. *Nonnodular parenchyma; ^#^Thyroid nodules.

Immunohistochemical analysis of TG, NQO1, and GPX2 was performed using thyroid tissues of the same patient with the L136P mutation, and FC values in the thyroid nodule were 1.04, 55.47, and 496.64, respectively (see Table 4 and Fig. 4). While TG expression in the nodule was equivalent to that in the nonnodular parenchyma, NQO1 and GPX2 expression was higher in the nodule than in the nonnodular parenchyma (Fig. 5B-5D). In the patient with multinodular goiter who did not harbor a *KEAP1* mutation, TG expression in the nodule was equivalent to that in the nonnodular parenchyma, while NQO1 and GPX2 expression was very low in both regions (Fig. 5F-5H). In the 4 probands with different *KEAP1* mutations, TG expression in the nodule was equivalent to that in the nonnodular parenchyma (Fig. 6A-6D), although varying degrees of nodule-dominant expression of NQO1 (Fig. 6E-6H) and GPX2 (Fig. 6I-6L) were detected.

## Discussion

To date, only 2 families with *KEAP1* germline mutations have been reported (3, 8); therefore, linking the phenotype of affected patients to genetic abnormalities is difficult. In this study, we present 5 families with *KEAP1* mutations, including 4 newly identified mutations. Their common histopathological features showed multinodular goiters located in the entire thyroid gland with few degenerative lesions, slow proliferation, and elevated expression of NRF2-targeted genes, specifically in the thyroid nodules.

Familial multinodular goiter caused by germline mutations often presents with coexisting tumors in multiple organs. For example, *DICER1* syndrome is accompanied by pleuropulmonary blastoma, ovarian Sertoli-Leydig cell tumor, or cystic nephroma (15), whereas *DGCR8* syndrome is complicated by schwannoma (4). While 2 family members with different *KEAP1* mutations had anaplastic thyroid and esophageal cancers (Q86* I-4 and L136P III-1), respectively, no frequent complications were detected in this study, suggesting a predisposition to thyroid-specific phenotypes by *KEAP1* germline heterozygous mutations. No other significant abnormalities were present in thyroid tissues, including driver gene mutations, although half the cases (2/4) had greater than 80% variant allele frequency of *KEAP1*, suggesting that the loss of heterozygosity of *KEAP1* may be partially involved in forming the thyroid nodules.

Significantly increased messenger RNA expression of many NRF2-targeted genes concerning antioxidant substances and detoxification in nodular lesions was noticed in a patient harboring a *KEAP1* mutation (p.L136P), compared to that in normal parenchyma. Immunohistochemistry confirmed the high protein expression of GPX2 and NQO1, representative NRF2-targeted genes, specifically in the thyroid nodules of various patients harboring *KEAP1* mutations. In contrast, TG (the precursor of the thyroid hormone) was too highly expressed in normal parenchyma to induce more in the nodule. The *Keap1*-null cell line derived from thyroid tissue showed a significantly higher expression of all GPX2, NQO1, and TG genes than the wild type (14). We identified nodule-specific upregulation of GPX2 and NQO1, but not TG, in the thyroid gland of patients with heterozygous germline mutations of *KEAP1*. We consider that NRF2 plays a central role in nodule formation in the thyroid gland with *KEAP1* mutations based on the nuclear accumulation of NRF2 by dissociation from mutant KEAP1 (p.R483H) in the cytoplasm (5, 8) and upregulation of its targeted genes in this study. Among the NRF2-targeted genes investigated using the RNA microarray, only prostaglandin-endoperoxide synthase 2 (PTGS2) expression was significantly reduced in the nodule (see Table 4). PTGS2, also called Cox-2, is a representative inflammatory cytokine and has a single consensus ARE sequence between nucleotides −562 and −572 of its promoter (16). Interestingly, heat stress upregulates antioxidant enzyme genes, such as *SOD1*, but downregulates *PTGS2* (17), indicating that although both genes have functional AREs, PTGS2 may develop responses different from antioxidant enzyme genes against various stress factors.

The thyroid gland generates high levels of reactive oxygen species (ROS) that are associated with thyroid hormone production. Minor ROS stress may be necessary for cellular homeostasis, while severe ROS induction leads to the apoptosis of thyroid cells by inducing mitochondrial and endoplasmic reticulum stress (18). In response to ROS stress, the expression of antioxidant substances induced by NRF2 protects against oxidative stress and confers multiple advantages to cell proliferation (7). In patients with *KEAP1* germline mutations, excessive NRF2 activity in the thyroid gland may exert a cytoprotective effect even against cells subjected to apoptosis by severe ROS generation, resulting in thyroid hyperplasia with scarce degradation. Of the 5 probands with *KEAP1* germline mutations, 1 had Graves disease with excessive hormone production, while the remaining 4 had normal thyroid function, indicating that normal hormone production is sufficient for developing thyroid hyperplasia.

This study had several limitations. First, there was a relatively small number of members with familial multinodular goiter who underwent genetic analysis. Second, thyroid specimens from patients with anaplastic thyroid carcinoma and esophageal cancer for further evaluation were absent. Third, comprehensive gene enrichment and functional annotation analyses using RNA microarray results were not possible because only one sample was available.

Collectively, our findings indicate that a germline heterozygous mutation of *KEAP1* is associated with the development of multinodular goiter as one pathogenesis. Genetic analysis of *KEAP1* should be considered in cases of familial multinodular thyroid nodules with an autosomal dominant mode of inheritance.

## Data Availability

Original data generated and analyzed during this study are included in this published article or in the data repositories listed in “References.”

## Abbreviations

Abs: antibodies
ARE: antioxidant response element
FC: fold change
FFPE: formalin-fixed paraffin-embedded
GPX2: human glutathione peroxidase 2
HE: hematoxylin-eosin
KEAP1: Kelch-like ECH-associated protein 1
NGS: next-generation sequencing
NQO1: human NAD(P)H quinone oxidoreductase 1
NRF2: nuclear factor erythroid-2–related factor 2
PTGS2: prostaglandin-endoperoxide synthase 2
ROS: reactive oxygen species
TG: human thyroglobulin
TPO: thyroid peroxidase
